# Femtosecond all-optical synchronization of an X-ray free-electron laser

**DOI:** 10.1038/ncomms6938

**Published:** 2015-01-20

**Authors:** S. Schulz, I. Grguraš, C. Behrens, H. Bromberger, J. T. Costello, M. K. Czwalinna, M. Felber, M. C. Hoffmann, M. Ilchen, H. Y. Liu, T. Mazza, M. Meyer, S. Pfeiffer, P. Prędki, S. Schefer, C. Schmidt, U. Wegner, H. Schlarb, A. L. Cavalieri

**Affiliations:** 1 Deutsches Elektronen-Synchrotron DESY, Notkestrasse 85, 22607 Hamburg, Germany; 2 Max Planck Institute for the Structure and Dynamics of Matter, Luruper Chaussee 149, 22761 Hamburg, Germany; 3 Center for Free-electron Laser Science (CFEL), Luruper Chaussee 149, 22761 Hamburg, Germany; 4 University of Hamburg, Luruper Chaussee 149, 22761 Hamburg, Germany; 5 SLAC National Accelerator Laboratory, 2575 Sand Hill Road, Menlo Park, California 94025, USA; 6 School of Physical Sciences and National Center for Plasma Science and Technology (NCPST), Dublin City University, Glasnevin, Dublin 9, Ireland; 7 European XFEL GmbH, Albert-Einstein-Ring 19, 22761 Hamburg, Germany; 8 Department of Microelectronics and Computer Science, Lodz University of Technology, ul. Wólczanska 221/223, 90-924 Łódź, Poland

## Abstract

Many advanced applications of X-ray free-electron lasers require pulse durations and time resolutions of only a few femtoseconds. To generate these pulses and to apply them in time-resolved experiments, synchronization techniques that can simultaneously lock all independent components, including all accelerator modules and all external optical lasers, to better than the delivered free-electron laser pulse duration, are needed. Here we achieve all-optical synchronization at the soft X-ray free-electron laser FLASH and demonstrate facility-wide timing to better than 30 fs r.m.s. for 90 fs X-ray photon pulses. Crucially, our analysis indicates that the performance of this optical synchronization is limited primarily by the free-electron laser pulse duration, and should naturally scale to the sub-10 femtosecond level with shorter X-ray pulses.

## Abstract (web summary)

Few-femtosecond synchronization at free-electron lasers is key for nearly all experimental applications, stable operation and future light source development. Here, Schulz *et al*. demonstrate all-optical synchronization of the soft X-ray FEL FLASH to better than 30 fs and illustrate a pathway to sub-10 fs.

## Graphical abstract

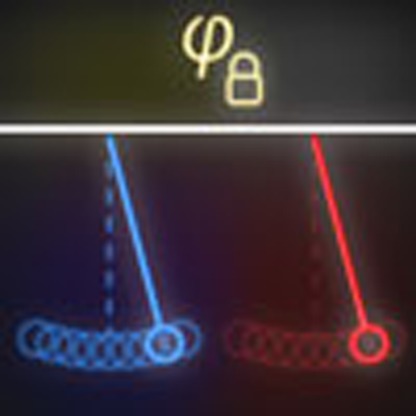

Large-scale free-electron laser facilities^1^,^2^,^3^,^4^ (FELs) are composed of many independent systems that are primarily synchronized using an electronic radio-frequency (RF) reference clock and electronic phase-locking techniques. A general measure of the overall facility-wide synchronization is given by the timing stability that can be maintained between the FEL X-ray pulses and independent external optical laser pulses. However, by relying solely on electronic phase-locking techniques, it has not been possible to synchronize these pulses to better than ~100 fs root mean square (r.m.s.)^5^,^6^,^7^,^8^, which prevents the use of FELs at their full potential and inhibits their future development.

Apart from the negative impact on experimental applications, insufficient synchronization between the accelerator subsystems adversely affects its internal operation and, consequently, FEL pulse generation. In conventional self-amplified spontaneous emission (SASE) FELs, short photon pulses with peak brilliance a billion times higher than can be reached at X-ray synchrotron sources are achieved through extreme compression of the driving electron bunch. However, the final compression depends on the energy and momentum distribution of the electrons within the bunch, which is more sensitive to the synchronization of the accelerating fields for stronger compression. As a result, critical FEL pulse parameters, including temporal profile, duration and intensity fluctuate from shot to shot, with increasing severity as shorter pulses are delivered.

The level of synchronization between the FEL and an external optical laser is critical in ultrafast experiments that exploit femtosecond X-ray pulses. Here, the optical laser pulse initiates the dynamics and the evolution of the system is probed with a subsequent FEL pulse. With insufficient synchronization, the delay between pump and probe cannot be precisely controlled and the maximum temporal resolution is limited by the timing instability, or jitter, between the two pulses rather than by the FEL pulse duration. Although measuring the relative arrival time of each pulse pair mitigates the problem by allowing for post processing^5^,^6^,^7^,^9^,^10^,^11^, these techniques can interfere with pump–probe experiments that are performed in parallel. Furthermore, this workaround is inefficient if the dynamics observed in the parallel experiment occur on a timescale that is shorter than the timing jitter, as, in this case, most measurements in the pump–probe experiment will occur outside the narrow time window of interest. In contrast, synchronization to better than the FEL pulse duration would enable most time-resolved pump–probe experiments at the highest possible resolution and would obviate the need for additional timing information. In addition, improved synchronization to this level would also stabilize the SASE FEL pulse parameters, expediting all experiments, including static biological imaging.

Femtosecond synchronization would also facilitate future development and application of both self-seeding and external FEL seeding schemes that can impose full control over the FEL pulse properties. In self-seeding, using a fixed spectral slice of the spontaneous X-ray emission for the seed, temporally coherent pulses are emitted allowing the FEL pulse duration to be tuned by varying the seed bandwidth^12^. Femtosecond synchronization would allow these pulses to be fully exploited in time-resolved applications. In addition, the severe intensity fluctuations that were observed during the initial demonstration of the self-seeding technique^13^ will be minimized, as the energy stability of the accelerator will be improved with better synchronization.

The FEL can also be seeded externally by overlapping an optical laser pulse with the driving electron bunch. In techniques based on high-gain high-harmonic generation^4^,^14^,^15^,^16^,^17^,^18^, or on echo-enabled harmonic generation^19^,^20^,^21^, FEL emission occurs at a harmonic of the seeding laser wavelength, and is fully coherent with temporal and spectral properties defined by the seed laser. External seeding directly at the desired FEL emission wavelength has also been demonstrated using optical lasers that are frequency-converted to extreme ultraviolet wavelengths by high-harmonic generation^22^,^23^,^24^,^25^. In pump–probe applications, externally seeded FELs ease synchronization requirements from a user’s perspective, as the seed and the FEL photon pulses are said to be intrinsically synchronized. However, facility-wide synchronization remains just as crucial, as it is required to ensure consistent temporal overlap between the seed laser pulse and driving electron bunch. In the future, further development of seeded FELs with controlled spectral phase will require femtosecond synchronization. Ultimately, a combination of these advanced techniques, external seeding and femtosecond optical synchronization will allow for pulse shaping in the spectral and temporal domain, having an impact on the emerging field of nonlinear X-ray science^26^,^27^,^28^,^29^,^30^.

To improve experimental conditions at existing facilities and enable future development of seeded FELs, we have implemented a synchronization system at FLASH, the free-electron laser in Hamburg, which is based on pulsed optical signals rather than electronic RF signals^31^,^32^. Although the individual elements of this system have been demonstrated in the past, our work further refines these techniques and combines them in a user FEL facility.

In this work, we demonstrate overall synchronization of (28±2) fs r.m.s. or (66±5) fs full width at half maximum (FWHM) while delivering (90±18) fs FWHM FEL photon pulses. Both parameters are simultaneously characterized by laser-based terahertz (THz) streaking spectroscopy. Furthermore, our analysis of the aggregate timing jitter in these experiments indicates that the optimum synchronization will improve automatically, as shorter electron bunches are used to generate even shorter femtosecond FEL pulses. The analysis leads us to conclude that the existing infrastructure at FLASH should support synchronization to below 10 fs. There is also no indication that this level of control cannot be achieved in complex, accelerator-based, SASE X-ray laser environments on the kilometre scale.

## Results

### Optical master laser oscillator

A high-purity reference signal must be distributed throughout the FEL facility to achieve femtosecond synchronization. To improve the reference at FLASH, the existing RF distribution systems, which are based on gigahertz (GHz) frequency electrical signals, were augmented with an optical timing distribution infrastructure. The optical reference signal is derived from the repetition rate of a femtosecond mode-locked laser oscillator. A fundamental advantage of this new reference is that it provides the possibility for temporal diagnostics based on mixing optical signals, which is inherently more precise than mixing electronic signals. Laser oscillators also exhibit superior short-term stability^33^,^34^, although they must still be cross-referenced to an electronic oscillator to maintain medium-term stability and to prevent long-term drifts^35^.

At FELs, the distance between subsystems can span hundreds of metres to kilometres and the distribution of any reference signal is particularly sensitive to environmental fluctuations caused by temperature and humidity changes, as well as mechanical vibrations. Therefore, the optical reference at FLASH is distributed on a fibre-optic network that is monitored using optical cross-correlation techniques. An optical cross-correlator is used to measure changes in the round-trip transit time for pulses sent through the network, allowing the effective fibre length to be determined with sub-100-as measurement precision^36^. Then, by introducing an adjustable optical delay the influence of the environmental fluctuations can be compensated. An additional independent cross-correlator is used to evaluate the network performance, and the network performance was found to be stable to better than 1 fs r.m.s. (see Methods for details on the implementation of the fibre network and the characterization measurement).

### Linear accelerator stabilization

**Figure 1:**
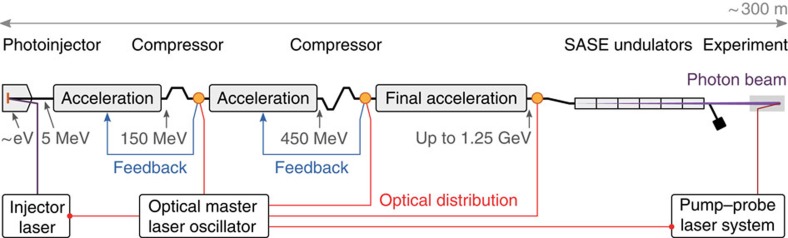
FLASH FEL facility. A macro-pulse of electron bunches is generated in a normal conducting photoinjector. Superconducting modules accelerate the bunches up to 1.25 GeV. Each bunch is compressed at intermediate energies of 150 and 450 MeV in magnetic chicanes. The arrival times of the electron bunches are measured with respect to the master laser oscillator after each compression stage and final acceleration (measurement stations indicated by orange dots in the schematic). The arrival times are incorporated in the feedback control loops for the amplitude and phase of the accelerating fields. The stabilized relativistic electron bunches are used to generate the SASE FEL pulses. Experiments can be carried out in conjunction with an external optical laser, which is synchronized to the master laser oscillator.

The FEL driving electron bunch is initiated with several electronvolts (eV) of kinetic energy at its source and requires hundreds of metres of linear accelerator systems to reach its final relativistic energy at the gigaelectronvolt (GeV) level. Shown schematically in Fig. 1, at FLASH a picosecond laser is used to generate the electron bunch by photoemission from the cathode in the injector. The photoelectrons are immediately extracted and accelerated to mitigate Coulomb repulsion and corresponding beam quality degradation. The energy is then further increased in RF accelerator modules. Once the electron bunch is relativistic, it can be compressed to achieve the final charge density required for SASE X-ray emission in the undulator.

At an FEL, the driving electron bunch is initially accelerated off-crest. As the head and tail of the bunch are subject to different accelerating gradients, a longitudinal energy chirp is introduced. Then, on propagation in a magnetic chicane, this correlated energy distribution leads to compression, as the path length or transit time of electrons within the bunch is energy-dependent. This process, however, is particularly sensitive to shot-to-shot fluctuation in the input electron bunch composition, which is caused by fluctuations in the amplitude and phase of the accelerating fields. These fluctuations also cause variation in the mean electron bunch energy, leading to instability in the overall transit time of the bunch through the compressor chicane resulting in the primary source of jitter with respect to the accelerator reference clock. Therefore, the beam arrival time jitter is a measure of the accelerator stability.

In the conventional approach to regulation, which is based solely on measurement of the fields inside the accelerating cavities^37^, typically stabilization of the arrival time of the electron bunches is limited to the 100-fs level. To improve conditions, the precise arrival time of electron bunches at the exit of the chicane must be incorporated in the accelerator feedback to better control the arrival time of subsequent bunches. With an optical reference signal, it is possible to measure the timing with ~5 fs r.m.s. measurement precision^38^ by mixing the transient electric field of the electron bunch with an optical pulse from the reference train in an electro-optic modulator^39^. Furthermore, as FLASH uses superconducting cavities, a burst of individual electron bunches with microsecond separation can be accelerated. The resulting macro-pulse of up to almost a millisecond in length has a repetition rate of 10 Hz. This pulse structure, combined with precise bunch arrival time measurements, is exploited to implement a beam-based feedback for greater control over the accelerator.

The performance of the beam-based feedback depends, in part, on how quickly the bunch arrival time can be evaluated, on the computation time required to calculate corrections in the accelerating fields and on the time required to transfer this information to the field controller. These factors are described by the latency of the feedback loop. With lower latency, corrections to the accelerating field can be applied faster, and stronger corrections can be made, resulting in a steady-state operation that is reached more rapidly with lower residual beam jitter. In addition to the computational limits, a physical limit on the speed and strength of the feedback is also imposed by the bandwidth of the accelerator cavity.

**Figure 2:**
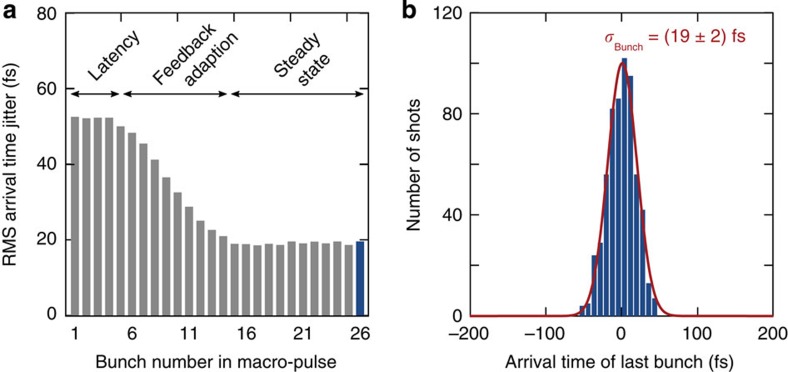
Beam-based feedback for accelerator stabilization. The width of the arrival time distribution for each bunch within the macro-pulse is evaluated over 600 consecutive single shots. As shown in **a**, the jitter is considerably reduced for bunches at the end of the macro-pulse where the accelerating fields have been corrected using the arrival time of the first bunches. The ‘latency’ period of this feedback spans the first 5 μs (5 bunches). During the ‘feedback adaptation’ period, the effect of the corrections reduces the observed jitter to the final steady-state value. The distribution of arrival times of the last bunch in the macro-pulse is shown in **b** and has a width of (19±2) fs r.m.s., with accuracy given by the numerical fit.

With the beam-based feedback engaged, the arrival time jitter for each of the bunches within the macro-pulse is measured over 600 consecutive shots, as shown in Fig. 2a. The jitter is largest for the first bunches and smallest for the last ones where the feedback has been applied. Although the arrival time jitter in the first bunch is >50 fs r.m.s., the jitter in the last has been reduced to (19±2) fs r.m.s. (where the error is determined from the Gaussian fit to the distribution). The arrival time distribution for the last bunch in the macro-pulse is shown explicitly in Fig. 2b. In practice, steady-state conditions are reached in only 15 μs (corresponding to 15 bunches for the macro-pulse used here). This performance is achieved through unique low-latency control algorithms, which are enabled by real-time calculations performed on integrated circuits with modern-field programmable gate arrays. Additional information concerning the operation and implementation of the bunch arrival time monitors, along with this beam-based control system, can be found in the Methods section.

### Optical locking of independent lasers at FELs

If the reference signal is distributed as a train of optical pulses from the master laser oscillator, the relative timing of an external laser can be measured directly using an optical cross-correlator. Then, by adjusting the external laser oscillator cavity length, its repetition rate can be tuned and stabilized, allowing the timing between the external laser and FEL to be controlled.

**Figure 3:**
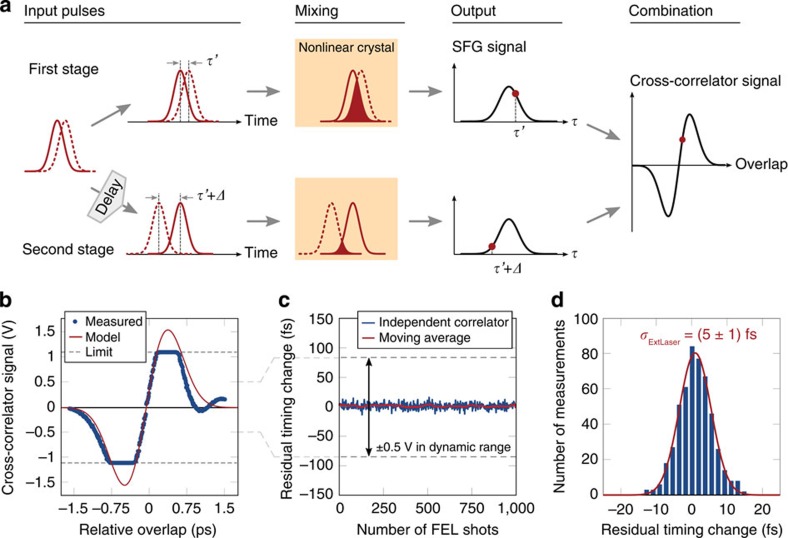
Optical locking of independent lasers. Optical cross-correlators measure the relative timing between two pulses. The principle of the device is illustrated in **a**. In the first stage, two input pulses with arbitrary timing are mixed in a nonlinear crystal resulting in an SFG signal with intensity that depends on their overlap. To determine which pulse arrived first, one of them is delayed by a fixed amount *Δ*, before they are overlapped again in the second SFG stage. The difference between the SFG intensities allows the exact input timing to be determined without sign ambiguity. A characteristic cross-correlator curve is traced, as illustrated, if the input timing is scanned continuously. A measured scan of the relative timing between the Ti:sapphire pump–probe laser and optical reference laser at FLASH results in the cross-correlator response plotted in **b**. Outside of the regions where the detector is limited (±1 V), the measured curve matches the curve calculated based on the input laser pulse durations. Once calibrated, the relative timing between the two pulses can be determined with sub-femtosecond accuracy within the ~400 fs dynamic range of the cross-correlator. Using this for feedback, the cavity length of the pump–probe oscillator is varied to lock the relative timing. The residual jitter between the external laser and reference is shown in **c**, as measured with an independent optical cross-correlator and is found to be (5±1) fs r.m.s., with accuracy given by the numerical fit to the corresponding distribution shown in **d**.

The measurement principle of an optical cross-correlator^31^,^40^ is shown in Fig. 3a. In the first stage, two pulses with arbitrary temporal overlap are mixed by sum-frequency generation in a nonlinear crystal. The strength of the mixed signal depends on the precise temporal overlap. To determine which pulse arrives first and to balance the cross-correlator, a second stage is used in which one pulse is delayed with respect to the other by a fixed amount. The rearranged pulses are mixed again, to generate another sum-frequency signal. By combining the two signals, the absolute delay between the pulses can be determined independently of intensity fluctuations, providing ideal feedback for locking the laser to the reference (see Methods for more details).

The external pump–probe laser oscillator at FLASH is synchronized to the accelerator using this scheme. Once the sources are locked, an independent optical cross-correlator is used for characterization. To calibrate the second cross-correlator, the relative timing between the stabilized reference and pump–probe oscillator is scanned across the full range of delays. The time dependence of the calibration signal is modelled using the input pulse durations as shown in Fig. 3b. Excluding the regions where the detector is limited (signal exceeding ±1 V), the measurement shows very good agreement with the expected values. The amplitude of the calculated cross-correlator signal can then be converted to provide the correct temporal offset for any measured voltage within the dynamic range with sub-femtosecond resolution.

Using this calibration, the performance of the optical synchronization is evaluated and the residual jitter was found to be (5±1) fs r.m.s., as shown in Fig. 3c,d. Here, the residual jitter is suppressed by an order of magnitude more than can be achieved in conventional electronic synchronization systems employed at FEL sources^41^,^42^. The improvement is due to the extreme sensitivity of the optical cross-correlator, which has a dynamic range of only ~400 fs. For comparison, with electronic signals the dynamic range of the feedback is typically several hundred picoseconds.

### Characterization of the complete synchronization system

**Figure 4:**
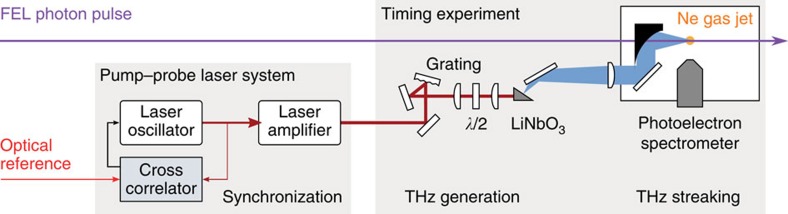
Relative timing between FEL and external laser. An external laser is optically locked to the accelerator reference clock signal and used to generate a single-cycle THz pulse by optical rectification in lithium niobate (LiNbO_3_). The resulting picosecond THz pulse and the FEL pulse are then overlapped in a neon (Ne) gas jet, where the FEL pulse profile and relative arrival time of the pulse with respect to the THz field is measured by streaking spectroscopy. As the THz pulse is phase-locked to the external laser, the arrival time with respect to the THz pulse is equivalent to the arrival time with respect to the external laser.

Single-shot, time-resolved photoelectron spectroscopy with single-cycle THz streaking fields is used to measure the FEL photon pulse duration as well as the relative arrival time between the FEL and amplified pump–probe laser pulses^7^. The experimental geometry is illustrated in Fig. 4. The FEL pulse is incident on a Ne gas target emitting a burst of photoelectrons that replicates its temporal profile. If the ionization occurs in the presence of a THz field, the final kinetic energy of the photoelectrons depends on the precise temporal overlap between the THz and FEL pulses. In this geometry, where the polarization of the field and direction of detection are parallel, the observed final kinetic energy (*E*_f_) as function of the instantaneous THz field strength at time 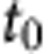 is, in atomic units, given by


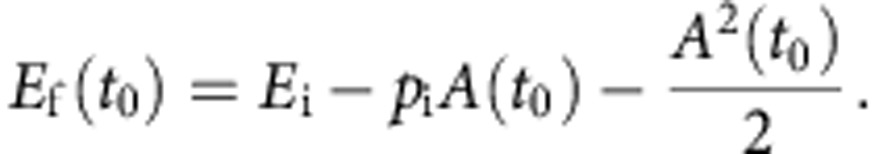


Here, *E*_i_ is the initial kinetic energy, *p*_i_ is the initial momentum and *A*(*t*_0_) is the vector potential of the THz field at the instant of photoionization^43^. When temporal overlap occurs on the single-valued streaking ramp of the THz vector potential, and the FEL pulse is shorter than the duration of the ramp, the arrival time and the temporal profile of the FEL pulse can be uniquely reconstructed from the measured photoelectron spectrum. As the THz pulses are generated by optical rectification of the amplified pump–probe laser pulse, the arrival time determined in the streaking measurement is equivalent to the relative arrival time between the FEL pulse and external amplified pump–probe laser.

**Figure 5:**
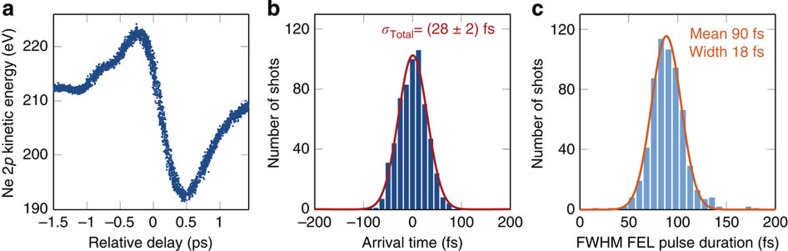
Facility-wide synchronization characterized by THz streaking. In **a**, the kinetic energy of the Ne 2*p* photoemission peak is plotted as the relative timing between the ionizing FEL pulse and the streaking THz pulse is scanned. Each data point corresponds to a distinct single-shot measurement. The final measured kinetic energy of the photoelectrons depends on the exact arrival time of the FEL pulse. Thus, by evaluating the position of the peak in the single-shot streaked photoelectron spectrum, the arrival time can be determined. Furthermore, the width of the streaked spectrum can be used to simultaneously determine the pulse duration. The arrival time distribution of 600 consecutive FEL pulses recorded at the zero-crossing of the streaking field is shown in **b**. The jitter is observed to be (28±2) fs r.m.s., with the error given by the numerical fit. The average pulse duration over these 600 consecutive shots was ~90 fs FWHM, with the corresponding distribution shown in **c**. The FWHM of the single-shot pulse duration was determined with an average measurement precision of 7 fs.

In the experiment, an electron bunch stabilized with beam-based feedback is used to generate the SASE FEL pulse with 234 eV photon energy. The centre-of-mass of the single-shot photoelectron spectra is used to define the average kinetic energy of the streaked photoelectrons. Figure 5a shows the change in kinetic energy of the streaked neon (Ne) 2*p* photoelectron peak, as the average delay between the THz streaking pulse and FEL pulse is varied continuously. The vector potential of the single-cycle THz pulse is clearly observed and, after averaging, this scan is used for *in-situ* characterization of the THz streaking field to calibrate the arrival time measurements (see Methods).

On the main streaking ramp, between −0.3 and 0.3 ps relative delay, the position of the photoelectron peak in kinetic energy is mapped to the arrival time with respect to the THz pulse, and therefore to the pump–probe laser. To evaluate the timing jitter, the desired relative delay is adjusted to time-zero where the measurement is most sensitive. Here, the single-shot arrival-time measurement precision is 3 fs r.m.s., limited primarily by fluctuation in the FEL photon energy, which causes an offset in the measured spectrum. The distribution of arrival times, collected over 600 consecutive single-shot measurements is shown in Fig. 5b and has a width of (28±2) fs r.m.s., where the error is determined from the Gaussian fit.

## Discussion

Simultaneous measurement of the FEL pulse duration provides context for the quality of the optical synchronization system. The distribution of FEL pulse durations, corresponding to the arrival time measurements described above, is shown in Fig. 5c. The FWHM of the X-ray pulse duration was determined on average with an accuracy of 7 fs, limited by the number of photoelectrons collected within the single shot. Crucially, the level of synchronization is better than the mean FEL pulse duration of 90 fs FWHM. Under these conditions, pump–probe experiments can be performed with near-optimal time resolution without additional arrival time experiments.

For more insight, the known sources of jitter can be isolated, allowing the remaining sources to be quantified. From independent measurements, the contribution due to the master laser oscillator and its optical distribution (*σ*_OptRef_) is determined to be ~1 fs r.m.s. The jitter due to the lock of the external laser oscillator to the optical reference (*σ*_ExtLaser_) is measured to be 5 fs r.m.s. Jitter of the electron bunch with respect to the optical reference (*σ*_Bunch_) is observed to be 19 fs r.m.s. However, as the measurement accuracy of these measurements was ~5 fs, on deconvolution the underlying beam jitter is determined to be 18 fs r.m.s. Assuming that the sources of jitter are uncorrelated, the total measured jitter is described as 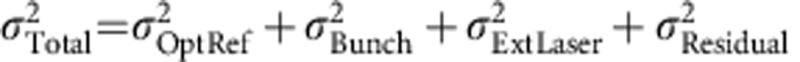, and the residual jitter (*σ*_Residual_) is ~20 fs r.m.s. under these particular machine operation parameters.

To reach the sub-10-fs level in overall synchronization, the beam jitter must be reduced and the remaining sources of residual jitter must be identified and minimized. Using the existing bunch arrival time monitors, it is expected that the electron bunch timing can be stabilized to the 5-fs level by further reduction of the latency in the feedback system. This will allow for increased feedback gain applied through optimized control algorithms using a broadband actuator.

The pump–probe laser amplifier may contribute to the residual jitter as only the oscillator seed pulse is synchronized to the optical reference clock. Fluctuations in the path length of the amplifier would then result in unaccounted timing instability. To eliminate this, the amplifier must be independently stabilized to the femtosecond level. In principle, this can be accomplished using similar optical cross-correlation techniques already applied throughout the synchronization network.

Based on the measured fluctuation of the FEL photon pulse duration, a more significant contribution to the residual jitter most probably results from instability in the composition of the compressed FEL-driving electron bunch. These instabilities change the regions of the bunch that satisfy the requirements for FEL emission from shot to shot. If the amplified FEL emission alternates between the head and tail of the electron bunch, additional jitter of up to tens of femtoseconds will be observed in the arrival time of the photon pulse^1^. However, as this effect scales with the electron bunch duration, it will naturally decrease as the electron bunch is further compressed to produce shorter photon pulses. Moreover, as the electron bunch is already compressed by a factor of ~50 from its initial generation at the photocathode, stronger compression has only a minor influence on the arrival time jitter of the electron bunch centroid. As FLASH is equipped with two magnetic chicanes for electron bunch compression, and the second acceleration section is operated moderately off-crest, arrival time jitter originating from the first acceleration stage is even slightly compressed in the second stage and is further reduced for stronger compression. Consequently, the overall arrival time jitter decreases. The required stability of the accelerating fields in the two stages can be provided by the beam-based feedback. Therefore, if fluctuations in the electron bunch are the dominant source of arrival time jitter of the FEL pulse, it should be possible to improve the overall synchronization to the sub-10-fs level for the shortest FEL pulses, eliminating the need for parallel arrival time measurements in nearly all applications.

In summary, an all-optical synchronization scheme was implemented at the FEL FLASH to control the arrival time of the FEL photon pulse and an external laser pulse to better than 30 fs r.m.s. A clear pathway towards sub-10-fs performance for a SASE-based FEL has also been identified. This level of synchronization, which has only been reported for an externally seeded FEL where intrinsic synchronization between the optical seed and the generated FEL pulses is expected^18^, will eliminate the need for additional arrival time measurements during pump–probe experiments and will promote the general stability of SASE sources, especially during short pulse operation. At self-seeded sources, which currently fluctuate in intensity by up to 100% due to spectral fluctuations in the initial SASE emission, better synchronization will improve the electron beam energy stability, ensuring useful seed radiation from shot to shot.

Femtosecond synchronization will also have an impact on future development of externally seeded sources, enabling new approaches that may even lead to the generation of attosecond FEL pulses at arbitrary X-ray photon energies. For example, by using a few-cycle carrier envelope phase-stablized laser pulse, an attosecond portion of the driving electron bunch could be isolated for FEL emission^44^. Alternatively, with femtosecond synchronization, proven high-gain harmonic generation techniques could be extended to enable advanced X-ray pulse shaping and temporal compression^45^. Finally, high-harmonic generation laser sources have recently been demonstrated at up to 1.6 keV^46^. With additional development and femtosecond synchronization, these sources could be suitable for direct seeding in the X-ray regime, presenting yet another pathway to the realization of intense, tailored attosecond X-ray pulses.

## Methods

### Two-colour optical cross-correlator

**Figure 6:**
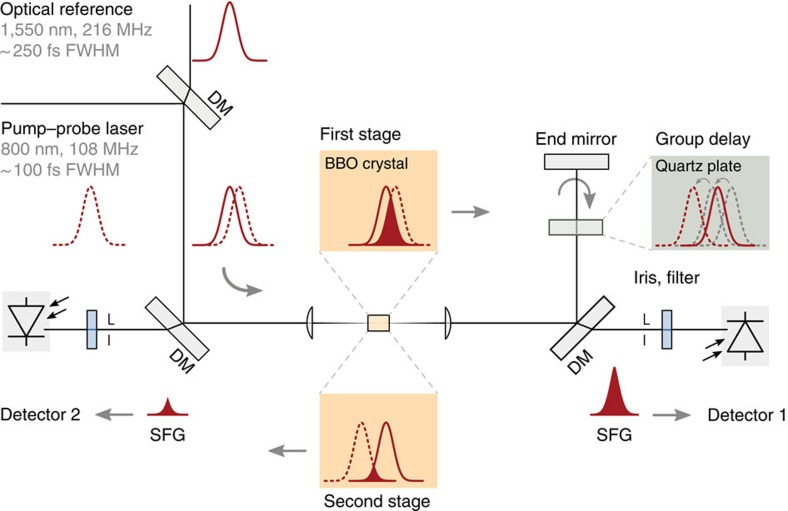
Schematic of a two-colour optical cross-correlator. The input pulses with arbitrary temporal overlap are combined with a dichroic mirror (DM) for collinear propagation and focused into a type-I phase-matched BBO crystal. In this ‘first stage’, the intensity of the SFG signal depends on the timing of the input pulses. The SFG signal is separated using another DM and measured with ‘detector 1’. The input pulses then pass through a quartz plate, are retro-reflected and pass the quartz plate again, so that one of the pulses is delayed with respect to the other by a fixed amount due to the different group velocities of the different wavelengths in quartz. When they are focused a second time back into the BBO crystal, another SFG signal is produced in the ‘second stage’ and measured with ‘detector 2’. Combination of the two signals gives the relative delay without sign ambiguity. The influence of amplitude fluctuations cancels out in a feedback loop, which keeps the temporal relation of the pulses fixed such that the combined signal is zero.

At FLASH, the pump–probe laser oscillator is locked to the optical reference clock using feedback from a two-colour optical cross-correlator. The experimental setup, adapted from previous work^31^,^40^, is shown in Fig. 6. First, the independent pulses of the optical reference and pump–probe laser oscillator with different carrier wavelengths are overlapped for collinear propagation with a dichroic mirror. The two pulses are then focused into a 500-μm-long type-I phase-matched *β*-barium borate (BBO) crystal where a sum-frequency signal is generated (sum-frequency generation, SFG). The intensity of the SFG depends on the temporal overlap and on the individual intensities of the input pulses. To monitor the SFG signal strength, it is separated from the input pulses with another dichroic mirror and measured with a first detector.

The SFG signal is symmetric as a function of relative delay; therefore, the same SFG signal strength is observed if the order of the two pulses is reversed. To eliminate this ambiguity, a second measurement is made after one of the pulses has been delayed with respect to the other by a fixed amount. To accomplish this, after the dichroic beamsplitter, the input pulses continue through a quartz plate, are retro-reflected and pass back through the quartz plate a second time. Propagation through the quartz plate introduces a fixed delay, because the group velocity is a function of wavelength. The optimal thickness of the quartz plate is defined by the pulse duration of the two lasers. As the optical reference and the pump–probe laser pulses have durations of ~250 and ~100 fs FWHM, respectively, the plate was chosen to be 6 mm thick. After delaying the pulses, they are re-focused back into the BBO crystal and another SFG signal is generated.

If the delayed pulse, which is identified by its carrier wavelength, was originally the first pulse entering the optical cross-correlator, the SFG signal from both stages of the device would be non-zero. If the delayed pulse had originally entered the device a second time, there would be no overlap in the second SFG stage and the signal strength would be zero. When the order of the pulses is correct, such that there is an SFG signal in both stages, small changes in the timing will lead to small changes in the SFG signal strength that have opposite sign in each stage. That is, an increase in signal strength in the first stage will correspond to a decrease in the second. Therefore, the direction of the change in relative timing can be determined unambiguously. Although the magnitude of the relative timing change is uncalibrated, this optical cross-correlator can still be used to maintain a fixed temporal overlap between the two laser pulses, if the overlap is chosen so that the sum-frequency signal from the two stages are equal. Then, as the timing between the two pulses fluctuates, feedback can be applied in the correct direction to compensate these fluctuations.

In our experiments, the combination of the sum-frequency signals from the first and second stage of the optical cross-correlator is performed using a differential amplifier (Femto DLPVA-100-B-D, 100 kHz bandwidth, 20 dB gain). In the optical cross-correlator, the average power of the 216 MHz optical reference signal is 7 mW, while the average pump–probe laser oscillator optical power is 15 mW at a repetition rate of 108 MHz. Together with a conversion efficiency of ~0.5% in the BBO crystal, an average SFG signal power of ~2 μW is generated. To increase the sensitivity, photomultipliers (Hamamatsu H6780, 40 dB gain, 10 kΩ load resistor) are used for detection.

In addition to the use as a source of direct and accurate feedback for the relative pulse timing, a second optical cross-correlator can be calibrated with the locked sources. The magnitude of the delay between a pair of pulses can then be measured with this calibrated optical cross-correlator. To convert a change in the cross-correlator output signal to a change in time, the relative pulse overlap between the stabilized sources is scanned with a motorized translation stage over all possible delays. Figure 3b in the Results section shows the characteristic optical cross-correlator signal, as the relative overlap between the two fundamental pulses is scanned over a total range of 3 ps. The measured trace is fit with a curve based on two Gaussian pulses with durations corresponding to the optical reference and the pump–probe laser. The calibration coefficient for measurements near the zero-crossing is approximated by the inverse slope of the fit at zero-crossing. Using this parameter, the lower detection threshold can be estimated from the detector baseline noise when no cross-correlation signal is present, that is, when there is no pulse overlap, and is found to be sub-100-as up to the detector bandwidth of 100 kHz.

The all-optical scheme improves on the timing sensitivity by two orders of magnitude compared with conventional electronic phase detectors, but has a limited dynamic range of ~400 fs in our present implementation. Therefore, the two laser sources are first coarsely locked before the all-optical synchronization is engaged, by using standard electronic phase detection. In this case, the dynamic range is given by half the RF accelerating reference period of 385 ps.

### Fibre-optic network and single-colour optical cross-correlator

**Figure 7:**
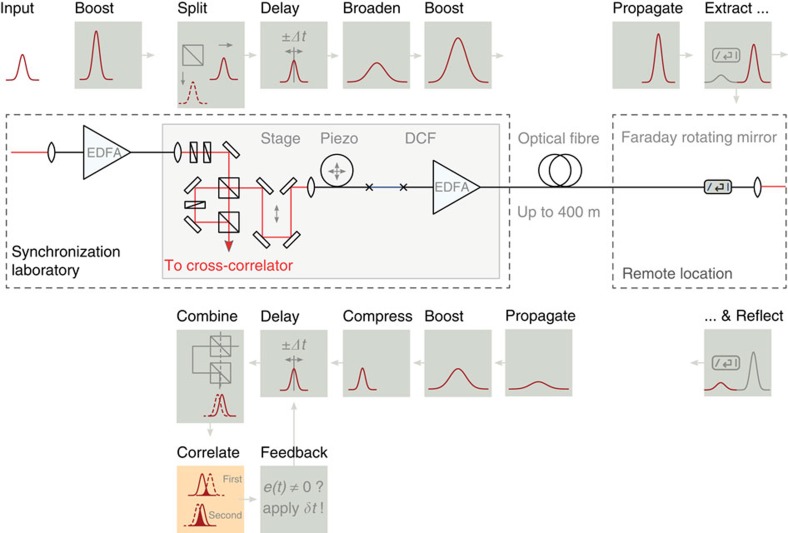
Fibre link stabilization. In the fibre link, reference pulses are initially amplified in an erbium-doped fibre amplifier (EDFA). Then, the pulse is split with a polarizing beamsplitter. One component is immediately coupled into the optical cross-correlator, while the other component is propagated down the fibre optic cable that runs along the accelerator tunnel to the remote end station. At the end station, a semi-transparent mirror and a Faraday rotator, which rotates the polarization of the pulse by 90°, partially reflects the pulse back to the entrance of the link. The timing of the back-reflected pulse with respect to a reference laser pulse that has not been propagated through the fibre is then measured in the optical cross-correlator. Changes in the relative timing, corresponding to changes in the transit time or length of the optical fibre, can be used as feedback to stabilize the length of the fibre link using a fast piezo-based fibre-stretcher (‘Piezo’) and a motorized optical delay line (‘Stage’). To accommodate for pulse broadening in the link, a dispersion compensating fibre (DCF) is incorporated.

At FLASH, the fibre-optic network used for the distribution of the optical reference signal is stabilized using single-colour optical cross-correlators^36^. Each end station, including the bunch arrival time monitors and the experiment pump–probe laser system, has an individual point-to-point connection to the master laser oscillator, with one shown schematically in Fig. 7. As the optical fibres in the accelerator tunnel are exposed to temperature and humidity changes, as well as mechanical vibrations, they must be length and therefore transit-time stabilized.

For feedback, the pulse is not only sent down the non-polarization maintaining fibre, but a fraction of it is retro-reflected in the same fibre using a semi-transparent mirror. In addition, a Faraday rotator is installed in front of this mirror. As the pulse passes through the rotator for the first time, its state of polarization is rotated by 45°. The reflected fraction of the pulse passes through the Faraday rotator a second time and its state of polarization is rotated by another 45°. Therefore, the polarization of the forward and backward travelling pulses is orthogonal. In practice, the Faraday rotator and the semi-transparent mirror are integrated into an all-fibre optic device. The orthogonal polarization of the two pulses allows the forward and back-reflected pulses to be separated and their relative timing can be evaluated in an optical cross-correlator that is based on second-harmonic generation in a type-II phase-matched, periodically-poled potassium titanyl phosphate (PPKTP) crystal.

In this cross-correlator, the temporal exchange of the two pulses, which is required for unambiguous detection, cannot be performed in quartz, as the two pulses being compared have the same wavelength. Instead, as the polarization of the two pulses is orthogonal, a birefringent crystal can be used to introduce the same effect. In fact, the temporal exchange is performed simultaneously with the second-harmonic generation in the PPKTP crystal. Furthermore, the exit face of the crystal is coated with a dichroic coating, which transmits the sum-frequency component but reflects the fundamental input pulses. Then, the second-stage SFG occurs during the retro-reflected pass of the pulses through the PPKTP crystal, eliminating the need for additional optical elements.

A fundamental limit on the bandwidth of this feedback is given by the fibre length, as one of the pulses must travel down the link and back before it can be mixed with an input forward-travelling pulse. As a result, only effects occurring on time scales longer than the round-trip time can be detected and compensated. The fibre length stabilization is realized with a fast piezo-based fibre stretcher (OptiPhase PZ2 with two layers, labelled ‘Piezo’ in Fig. 7). In practice, the maximum speed of the piezo sets another limit on the bandwidth of the feedback. When the piezo-based actuator reaches the end of its range, which is±4 ps, a motorized optical delay line is moved such that the operation point of the stretcher is centred again. In total, a delay of >1 ns is covered for the fibre links installed at FLASH.

Dispersive pulse broadening at the fibre link end and in the optical cross-correlator is compensated by including a chromatic dispersion compensating fibre in the link. In addition, to boost the pulse energy in the cross-correlator and for use at the end station, two erbium-doped fibre amplifiers are incorporated.

**Figure 8:**
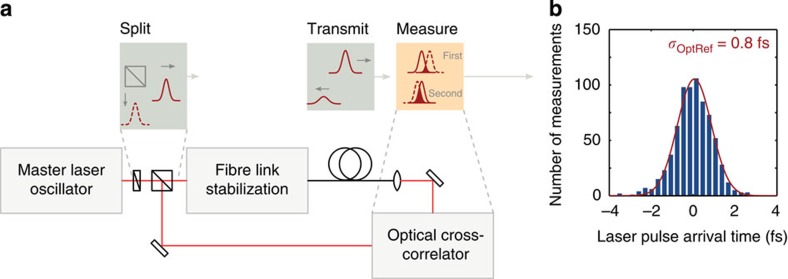
Fibre link characterization. The distribution of relative arrival time is compiled from measurements between laser pulses extracted from the fibre link and pulses directly from the master laser oscillator, which were made with an independent out-of-loop optical cross-correlator, as shown in **a**. Each measurement gives the average temporal overlap over an interval of 2 ms and measurements were made every 5 s for 1 h. Over this period, a residual timing jitter of 0.8 fs r.m.s. was observed, determined from the Gaussian fit to the corresponding distribution shown in **b**.

To characterize the performance of the fibre link stabilization scheme, a test device was commissioned with a 400-m-long fibre through the accelerator hall but returning back to the lab, where the master laser oscillator is installed, as shown in Fig. 8a. The optical pulses were extracted from the fibre link end and measured directly with respect to optical reference pulses in an independent calibrated cross-correlator. The fluctuations of the relative laser pulse arrival time in this test were measured to be <1 fs r.m.s. over a 1-h interval, with a distribution shown in Fig. 8b.

### Electron bunch stabilization

At FLASH, there are two major feedbacks involved in the stabilization of the accelerating fields and, ultimately, the delivery time of the electron bunch at the undulator structure. First, the accelerating fields inside the cavities are measured and fluctuations are corrected. For these measurements, an antenna is installed directly in the superconducting accelerator modules. Using this technique of self-regulation, the accelerating field can be controlled with a closed-loop bandwidth of 40 kHz, limited by the intrinsic bandwidth of the superconducting cavities and the maximum possible gain applicable in the feedback loop. Under optimum conditions, this allows the electron bunch timing jitter to be stabilized to 50 fs r.m.s. at FLASH, measured with respect to the optical reference. Uncorrected, remaining RF field amplitude and phase fluctuations lead to energy fluctuations in the accelerated electron bunch, which changes the trajectory and transit time through the bunch compressor section.

To further improve the stabilization, the actual arrival time and the compression state of the electron bunch can be measured and included in the feedback loop. Within the optical synchronization network, femtosecond-precision measurement of the arrival time of the relativistic electron bunch relies on mixing its associated transient transverse electric field with an optical reference laser pulse. To collect the field of the electron bunch, a pair of antennas^47^ is mounted symmetrically around the electron beam path. When the bunch passes, its transverse electric field induces a fast transient electronic signal with several GHz bandwidth in each antenna. To eliminate the influence of variation in the beam orbit, the signals from the pair of opposing antennas are combined. The resulting signal can then be applied to an electro-optic modulator for mixing with the reference laser pulse.

**Figure 9:**
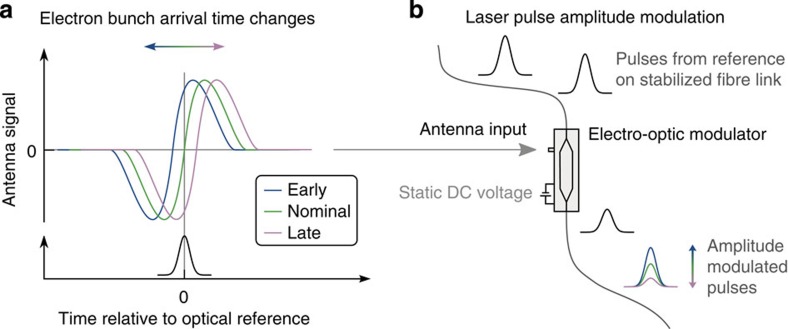
Bunch arrival time measurement. The relativistic electron bunch induces a bipolar transient signal in an antenna, as shown in **a**. Together with pulses from the optical reference, this signal is coupled into an integrated electro-optic modulator, as sketched in **b**. The degree of modulation depends on the temporal overlap between the electric signal and the laser pulse, imprinting the arrival time of the electron bunch onto the laser pulse amplitude, which is subsequently measured in a real-time balanced detection scheme.

The all-fibre electro-optic modulator is built directly into the optical fibre distribution network eliminating the need for additional optics and associated instabilities. The photonic circuit is built in a Mach–Zehnder geometry, where the laser pulse is split into two arms. In each arm, the laser pulse experiences a phase delay depending on the transient electric signal from the antennas. Then, on recombination, the laser pulses from each arm interfere depending on the phase shift incurred in the modulator. Initially, the transmission through the device is set to 50% by applying a static, direct current (DC) voltage to the electro-optic modulator. Then, with the addition of the transient electric field associated with the electron bunch, the amplitude of the transmitted pulse is increased or decreased, depending on the relative timing between the laser pulse and the electron bunch (see Fig. 9, adapted from ref. 48). The dynamic range of the measurement is several picoseconds, given by the temporal structure of the signal from the beam antenna. Notably, the electronic signal generated in the antenna is not sensitive to variation in the phase-space of the compressed electron bunch, and therefore only the relative arrival time of the centre-of-mass of the bunch charge distribution with respect to the optical reference pulse can be measured. To minimize the influence of fluctuations in the laser pulse amplitude that could falsely indicate timing changes, a series of reference measurements is made for comparison^49^. Two measurements are made on the background signal level when there is no optical pulse to establish the noise floor of the measurement. Another measurement is made on a reference pulse that is not affected by the electron bunch to establish the amplitude of the unmodulated transmitted pulse. Finally, a measurement is also made on the pulse that overlaps with the bunch transient in the electro-optic modulator. Combining the four measurements provides an accurate measure of the transmission and timing. To calibrate the device, allowing the level of transmission to be mapped to its corresponding arrival time, the optical reference pulse is scanned through several picoseconds of relative delay with respect to the electron bunch. A linear slope is then fit to the averaged data set.

The accuracy of the single-shot arrival time measurement is determined by observing the arrival time of a single electron bunch using two independent monitors separated by the final, straight, non-dispersive section of the accelerator (see Fig. 1 in the Results section). Under the conditions present during the experiment, the accuracy of the bunch arrival time monitor was ~5 fs r.m.s., as determined by correlation analysis of the independent arrival time measurements in the accelerator. This level of accuracy includes timing jitter in the reference clock distribution network.

In addition, a measurement of the compression is made using a dedicated bunch compression monitor^50^ located adjacent to the arrival time monitors in the accelerator. These are based on pyroelectric detection of diffraction radiation and are not directly connected to the fibre-optic synchronization system.

Finally, the measurements of the electron bunch arrival time and the compression are combined in the feedback controller, and used to calculate the corrections for amplitude and phase, as the two degrees of freedom of the accelerating fields.

### THz photoelectron streaking

Single-cycle THz pulses for time-resolved photoelectron streaking spectroscopy were generated by optical rectification of ~60 fs FWHM, ~3.5 mJ Ti:sapphire near-infrared pulses, delivered by the FLASH pump–probe laser system, in lithium niobate (LiNbO_3_) using the tilted pulse front method^51^. After generation, the THz pulse was transported into the vacuum chamber and focused in the interaction region for the streaking measurement using a telescope consisting of a 300-mm collimating transmission Teflon lens and a 150-mm focal length off-axis parabolic mirror (see Fig. 4 in the Results section).

The THz pulse was polarized horizontally, parallel to the FEL photon pulse polarization, which is fixed by the undulator geometry. The time-of-flight spectrometer was also mounted horizontally. In this orientation, the THz streaking effect acts directly on the measured photoelectron kinetic energy. Furthermore, the photoemission is peaked in the horizontal plane due to the polarization of the FEL photon pulse, leading to maximum signal.

To evaluate the facility-wide timing jitter at FLASH and to determine the FEL pulse duration, a calibrated map must be constructed to transform the measured streaked photoelectron spectra from kinetic energy to time. In these measurements, as the jitter between the FEL and THz pulses was small in comparison with the THz pulse duration, this map can be retrieved directly from a set of streaking measurements made as the average delay was scanned continuously over the full range of the pulse. Compiling the map in this way eliminates uncertainty that could be introduced by sampling a portion of the THz focus that does not interact with the FEL pulse in the gas target.

**Figure 10:**
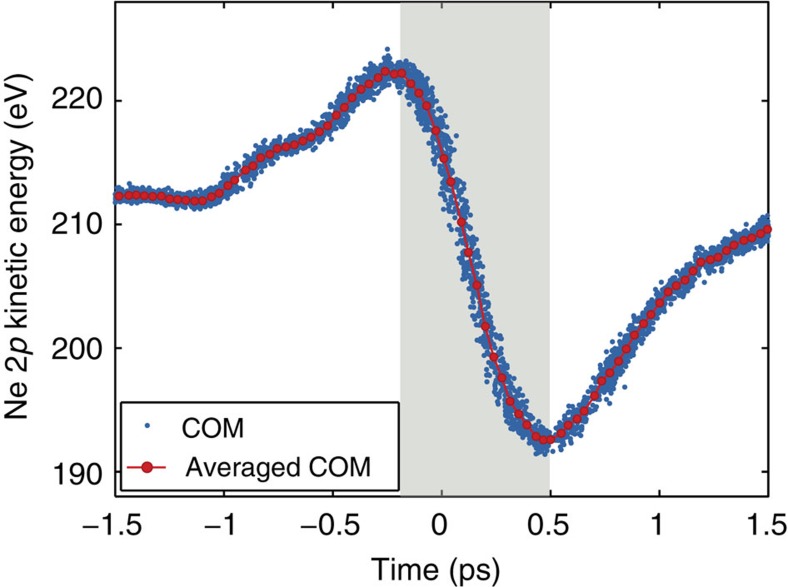
Streaking map. The centre-of-mass (COM) of the Ne 2*p* photoelectron peak is shifted in kinetic energy depending on the temporal overlap of the ionizing FEL pulse and the streaking THz pulse. As the sources are closely synchronized, the shift in kinetic energy as a function of temporal overlap can be accurately mapped by averaging the shift over several single-shot measurements as the relative delay is scanned. Interpolating the averaged, shifted COM at each delay results in a continuous curve that can be used to map the exact arrival time of any streaked photoelectron spectrum generated by an FEL pulse that arrives within the ~720-fs, single-valued streaking ramp (indicated by the shaded region in the plot).

To retrieve the map directly from the set of streaking measurements, the centre-of-mass of each streaked single-shot spectrum was calculated and plotted versus the set delay where the measurement was recorded. The full set of measurements is composed of ~5,000 single shots collected over 3 ps of relative delay. The acquired data was then binned in time and interpolated to generate the transformation map plotted in Fig. 10. The furthest upshifted and downshifted single-shot photoelectron spectra were found at 225 and 190 eV, respectively, corresponding to a THz pulse with a peak electric field of 85 kV cm^−1^.

When the FEL photon pulse overlaps at or near the zero crossing of the streaking field, the pulse duration can be retrieved by first fitting each streaked photoelectron spectrum with a Gaussian curve. Next, the 2.7-eV FWHM energy resolution of the time-of-flight spectrometer is deconvolved from the Gaussian fits. This allows the broadening observed in the streaked spectrum, due to the length of the FEL pulse, to be isolated. The deconvolved width in kinetic energy is then converted to femtoseconds using the gradient of the THz streaking field.

To evaluate the accuracy of this pulse duration measurement, a similar procedure to that used in past experiments^8^ is applied. In this procedure, the statistical error in the single-shot photoelectron spectra is determined by counting the number of detected photoelectrons within the energy resolution window of the detector by boxcar integration centred at each collected data point. As the streaked spectra are heavily oversampled (300 data points were collected within the ~35-eV streaked kinetic energy range), an error envelope for the measured spectrum is generated rather than discrete points with individual error bars. The photoelectron spectrometer energy resolution is then deconvolved from the upper and lower bound of the error envelope. By fitting the envelope and converting to time, as described above, the accuracy of each single-shot measurement can be determined. For the 600 measurements evaluated in the Results section, on average the accuracy of the recovered FEL pulse duration is determined to be (90+6/−7) fs FWHM.

## Author contributions

I.G., C.B., M.C.H., M.I., H.Y.L., T.M., H.B., U.W., S.S. and A.L.C. performed the THz streaking characterization of the overall synchronization. S.S., M.K.C., M.F. and H.S. implemented the reference optical master laser oscillator and its distribution network. M.F., P.P., S.Schefer, U.W. and S.S. synchronized the pump–probe laser. M.K.C., S.P., S.S., C.S. and H.S. implemented the beam-based accelerator stabilization. I.G., S.S. and A.L.C. analysed and interpreted the data. S.S., I.G., C.B., J.T.C., M.M., H.S. and A.L.C. wrote the manuscript.

## Additional information

**How to cite this article:** Schulz, S. *et al*. Femtosecond all-optical synchronization of an X-ray free-electron laser. *Nat. Commun.* 6:5938 doi: 10.1038/ncomms6938 (2015).
